# Targeted amplification for enhanced detection of biothreat agents by next-generation sequencing

**DOI:** 10.1186/s13104-015-1530-0

**Published:** 2015-11-16

**Authors:** Shea N. Gardner, Kenneth G. Frey, Cassie L. Redden, James B. Thissen, Jonathan E. Allen, Adam F. Allred, Matthew D. Dyer, Vishwesh P. Mokashi, Tom R. Slezak

**Affiliations:** Bioinformatics, Global Security Program, Lawrence Livermore National Laboratory, 7000 East Avenue, L-174, Livermore, CA 94550 USA; Naval Medical Research Center, NMRC-Frederick, 8400 Research Plaza, Fort Detrick, MD 21702 USA; Henry M. Jackson Foundation, 6720-A Rockledge Drive, Suite 100, Bethesda, MD 20817 USA; Thermo Fisher Scientific, 180 Oyster Point Boulevard, Building 200, South San Francisco, CA 94080 USA

**Keywords:** Targeted amplification, Biodefense, Next Generation Sequencing

## Abstract

**Background:**

Historically, identification of causal agents of disease has relied heavily on the ability to culture the organism in the laboratory and/or the use of pathogen-specific antibodies or sequence-based probes. However, these methods can be limiting: Even highly sensitive PCR-based assays must be continually updated due to signature degradation as new target strains and near neighbors are sequenced. Thus, there has been a need for assays that do not suffer as greatly from these limitations and/or biases. Recent advances in library preparation technologies for Next-Generation Sequencing (NGS) are focusing on the use of targeted amplification and targeted enrichment/capture to ensure that the most highly discriminating regions of the genomes of known targets (organism-unique regions and/or regions containing functionally important genes or phylogenetically-discriminating SNPs) will be sequenced, regardless of the complex sample background.

**Results:**

In the present study, we have assessed the feasibility of targeted sequence enhancement via amplification to facilitate detection of a bacterial pathogen present in low copy numbers in a background of human genomic material. Our results indicate that the targeted amplification of signature regions can effectively identify pathogen genomic material present in as little as 10 copies per ml in a complex sample. Importantly, the correct species and strain calls could be made in amplified samples, while this was not possible in unamplified samples.

**Conclusions:**

The results presented here demonstrate the efficacy of a targeted amplification approach to biothreat detection, using multiple highly-discriminative amplicons per biothreat organism that provide redundancy in case of variation in some primer regions. Importantly, strain level discrimination was possible at levels of 10 genome equivalents. Similar results could be obtained through use of panels focused on the identification of amplicons targeted for specific genes or SNPs instead of, or in addition to, those targeted for specific organisms (ongoing gene-targeting work to be reported later). Note that without some form of targeted enhancement, the enormous background present in complex clinical and environmental samples makes it highly unlikely that sufficient coverage of key pathogen(s) present in the sample will be achieved with current NGS technology to guarantee that the most highly discriminating regions will be sequenced.

**Electronic supplementary material:**

The online version of this article (doi:10.1186/s13104-015-1530-0) contains supplementary material, which is available to authorized users.

## Background

Nucleic acid based methods of pathogen detection, such as PCR, although robust and sensitive, are often subject to false positive and false negative readings. False positive errors are often due to detection of a closely related near neighbor species, a trend observed with biothreat agents [1, 2]. False negative errors are frequently the result of levels of target nucleic acid at or below the sensitivity threshold [3]. Additionally, strain variation at the nucleotide level can lead to signature erosion. This is especially true in organisms with high mutation rates [4]. Although PCR-based assays are capable of strain designation, this often requires design of multiple primer and probe sequences [5]. Additionally, the number of primer pairs necessary to detect a large set of organisms or strains is often untenable or requires multiple rounds of primer design, complex bioinformatic software or both [6].

High-throughput next generation sequencing (NGS) has become a viable solution to many of these problems. Detection of pathogens by NGS is not hampered by the constraints discussed above. NGS is relatively unbiased, not dependent on specific signature sequence information, and requires no a priori knowledge about a pathogen. In addition, NGS is culture-independent and requires only high-quality nucleic acid. However, NGS is not without drawbacks. Until recently, one of the major impediments to using NGS for point-of-need (PON) detection/diagnostics has been a large amount of time (≥7 days) from sample to answer as well as the technical training and experience necessary to produce quality sequence reads. In addition, NGS has conventionally required nucleic acid masses at least an order of magnitude greater than a PCR-based assay. Finally, the laboratory space requirements of the sequencer as well as the required ancillary equipment often precluded the use of NGS by all but the largest research groups.

The advent of benchtop sequencers with easy to use and streamlined workflows, such as the Ion Torrent™ Personal Genome Machine™, has alleviated many of these concerns. A number of recent clinical studies have shown the utility of benchtop NGS in identification and attribution of disease-causing microorganisms. Specifically, NGS was used successfully to track nosocomial infection of soldiers returning from Afghanistan. In this case, standard typing techniques failed to distinguish between isolates, while NGS uncovered a number of single-nucleotide polymorphisms (SNPs) that allowed investigators to establish an epidemiologic chain [7]. More recently, using NGS, two groups were simultaneously able to identify the causative agent of an outbreak of enterohaemorrhagic *Escherichia coli* (EHEC) in patients in a National Institutes of Health (NIH) clinical ward. Another study showed the ability of NGS to rapidly and precisely identify outbreak clusters of MRSA and *Clostridium difficile* [8]. However, these studies were performed with clinical isolates of the microbe in question, not by metagenomic sequencing of complex primary samples.

Multiple studies have documented the difficulties of current NGS platforms to yield confident species identification for microbial agents in complex backgrounds [9, 10]. Most data to date from these projects indicate that the current NGS platforms using unbiased sequencing simply do not provide enough pathogen coverage over the background to provide even species-level identification. Simply put, bench-top NGS cannot sequence deeply enough into a complex environmental or clinical trace-level sample to provide even confident species identification, much less strain identification or presence of specific known resistance/virulence factors [11, 12]. While NGS instruments like the Illumina^®^ HiSeq can produce billions of reads, their long run times (i.e. >7 days) preclude obtaining actionable information quickly enough to guide a response to a public health crisis. Additionally, biases in read coverage result in areas of the genome with low, or no coverage, a trend that is prominent in AT-rich areas [13]. Finally, a large proportion of unbiased sequence reads will represent the ‘core genome’ of shared elements found in both pathogenic and benign organisms of closely related taxa. For instance, a study of an important zoonotic pathogen, *Streptococcus suis,* indicated that 876 genes comprised a Minimum Core Genome (MCG). Of medical significance, only 9 of 21 known virulence genes were shared among all strains of this species. Similarly, core genome estimates for several other organisms of medical interest ranged from 42.7 % (*S. pneumoniae*) to 15.5 % (*H. pylori*). It is important to note that these studies identified core elements of clonal isolates *within* a species [14, 15]. Thus, enhancement strategies that increase our ability to discriminate between closely-related organisms are needed.

There are a number of wet lab methods that aim to ameliorate this shortcoming of highly informative sequence reads. In general, enhancement strategies employ one of two tactics: targeted amplification or sequence-based capture enrichment. A familiar and widely-employed technique using target amplification is 16S rRNA gene sequencing [16]. In this approach, universal primers are used to amplify conserved regions of the 16S gene [17]. This method has been utilized in numerous studies of bacterial communities (reviewed in [18]). Alternatively, investigators have made use of capture-based enrichment techniques to enrich a sample for sequences of interest. In one particular example, samples can be depleted of ribosomal RNA using probes bound to solid-state supports [19].

However, these methods are not without limitations. Although 16S sequencing is widely accepted and constitutes a current ‘gold standard’ in molecular typing, it has low resolution and it has been shown that primer selection can introduce bias in the resultant amplicons [20]. Sequence capture enrichment techniques are subject to long hybridization times and generally require probes that far exceed the length of PCR primers [21]. Moreover, the bulk of capture-based enrichment technologies have emphasized the human genome (reviewed in [22]) rather than microbial pathogens. More recent development efforts have focused on use of multiplex PCR prior to sequencing. For instance, micro-droplet PCR has been shown to enable sequencing of nearly 4000 amplicons [23]. A number of studies have evaluated the efficacy of multiplex sequencing to characterize human disease as well as microbial strain differentiation [24–26]. Specifically, Life Technologies’ (now Thermo Fisher Scientific) Ion AmpliSeq™ technology has been used for human SNP typing [25] and detection of genetic variation in human cardiomyopathy [26] among others. Ion AmpliSeq™ technology currently supports up to 6144 custom primer pairs for targeting custom regions. We have utilized the Life Technologies Ion AmpliSeq™ robust end-user customization capability to support other markets, such as pathogen detection, in addition to their standard exon and cancer biomarker panels.

Since no commercial panel for biothreat detection exists, we sought to design and test a custom AmpliSeq™ pilot panel targeting a limited number of microbial agents. For this proof of concept study we focused on organisms of concern to biodefense and public health, specifically microbes from CDC Bioterrorism Tier 1 (*B. anthracis*, *Y. pestis, F. tularensis*) and Tier 2 (*B. pseudomallei*). In addition to determining the efficacy of our custom panel, we investigated the analytical sensitivity of the AmpliSeq™ protocol as applied to our mock clinical samples.

## Results

### Comparison of fragment libraries versus AmpliSeq™ libraries

**Fig. 1:**
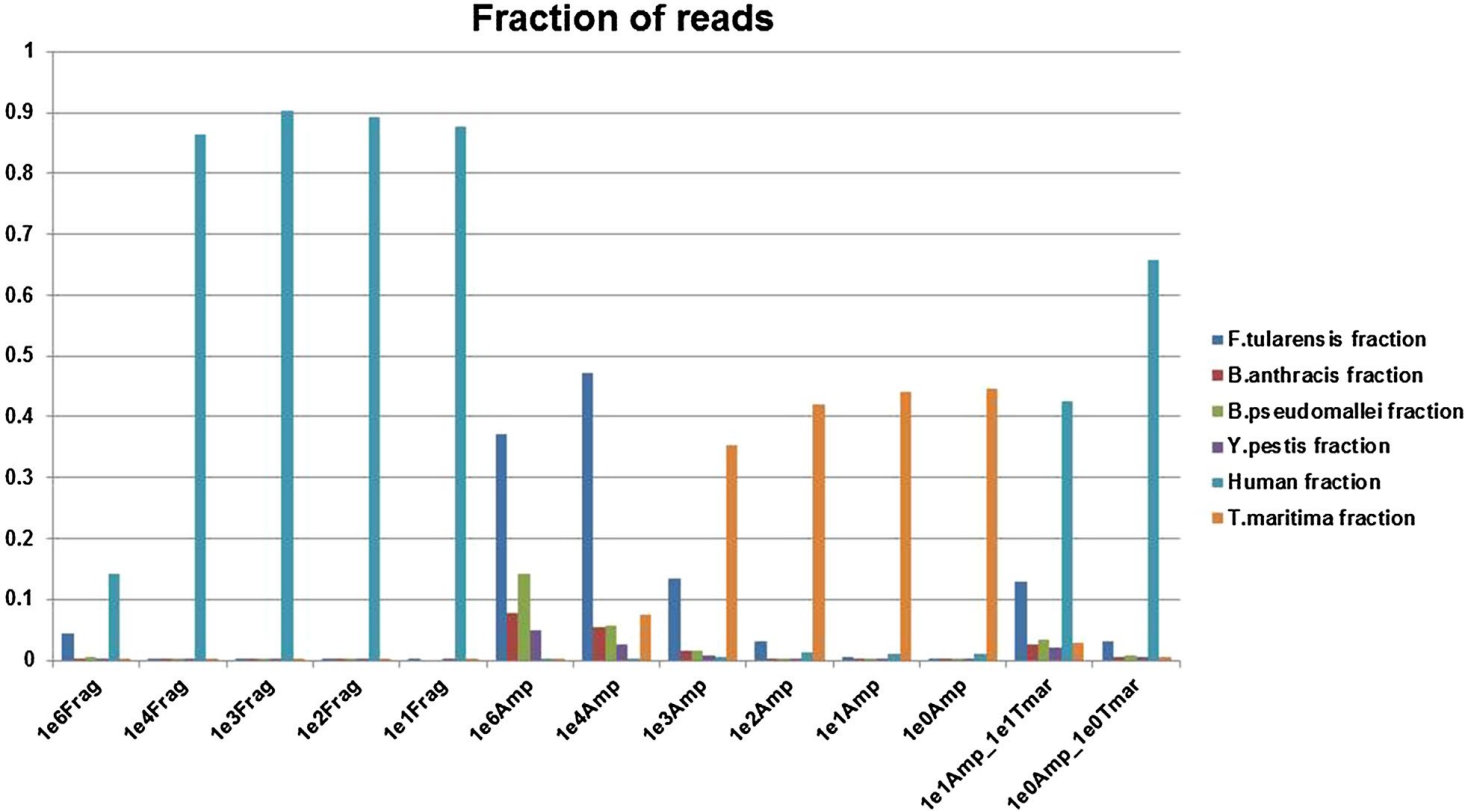
Fraction of reads classified at indicated taxa. Sequence reads resulting from the indicated samples were classified using LMAT with the complete genome database. Numbers are expressed as fraction of the total reads in each particular run. See Additional file 1: Figure S1, Additional file 2: Figure S2, Additional file 3: Figure S3, Additional file 4: Figure S4 for organism-specific details

In every case, the AmpliSeq™ libraries demonstrated a sizable increase in the number of pathogen-specific reads with a concomitant reduction in human reads as compared to fragment libraries prepared from the same samples (Fig. 1). Specifically, the ratio of reads classified as human declined from as high as 0.9 in the fragment libraries to less than 0.1 in 6 of 8 AmpliSeq™ libraries (Compare 1e3 fragment library with 1e3 AmpliSeq library). For each of the four spike-in organisms, we noted an increase of at least 1 order of magnitude in pathogen reads in the AmpliSeq™ libraries when compared with the corresponding fragment library. This observation was especially striking in the 1e2 genomic equivalents (GE) samples (Additional file 1: Figure S1, Additional file 2: Figure S2, Additional file 3: Figure S3, Additional file 4: Figure S4). For instance, reads specific to *B. pseudomallei* improved from less than 100 in the fragment library to over 10,000 in the AmpliSeq™ library (Additional file 2: Figure S2).

**Fig. 2:**
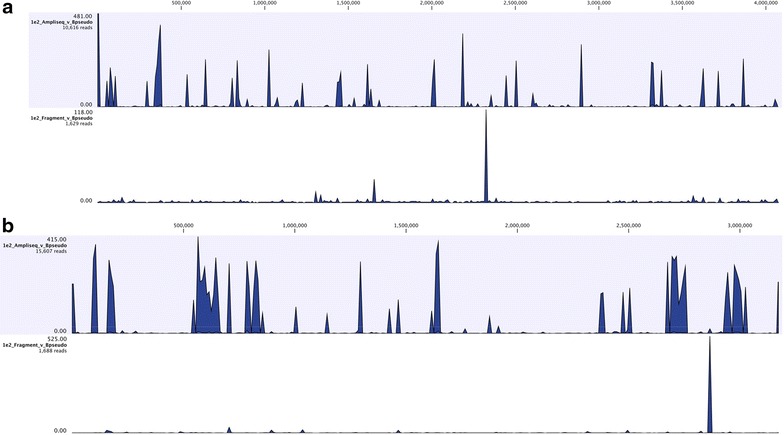
Read mapping against *B. pseudomallei* K96432 at 1e2 spike-in. **a** 10,616 reads from an AmpliSeq library mapped to Chromosome 1 of *B. pseudomallei* K96432 (*top*) compared with 1629 reads from a Fragment library (*bottom*). **b** 15,607 reads from an AmpliSeq library mapped to Chromosome 2 of *B. pseudomallei* K96432 (*top*) compared with 1688 reads from a Fragment library (*bottom*)

Specifically, at a spike-in level of 100 GE for *B. pseudomallei*, the fragment library yielded a total number of reads that was at least one order of magnitude less than that for the AmpliSeq library (Fig. 2a, b). Although reads from the fragment library were spread over both chromosomes, the depth of coverage was low (<10×). In contrast, reads from the AmpliSeq libraries were present at high coverage levels (>100×). Note that the AmpliSeq™ libraries result in species-specific reads distributed throughout the genome (Fig. 2a, b).

No reads corresponding to the spike-in organisms were present in fragment libraries at a spike-in level of less than 100 GE. Importantly, at spike-in levels corresponding to 10 and 1 GE, AmpliSeq™ libraries produced informative species-defining reads from all spike-in organisms. Conversely, fragment libraries were dominated by the human background. Finally, no sequence reads were classified as *S. enterica,* which served here as a negative amplification control.

**Fig. 3:**
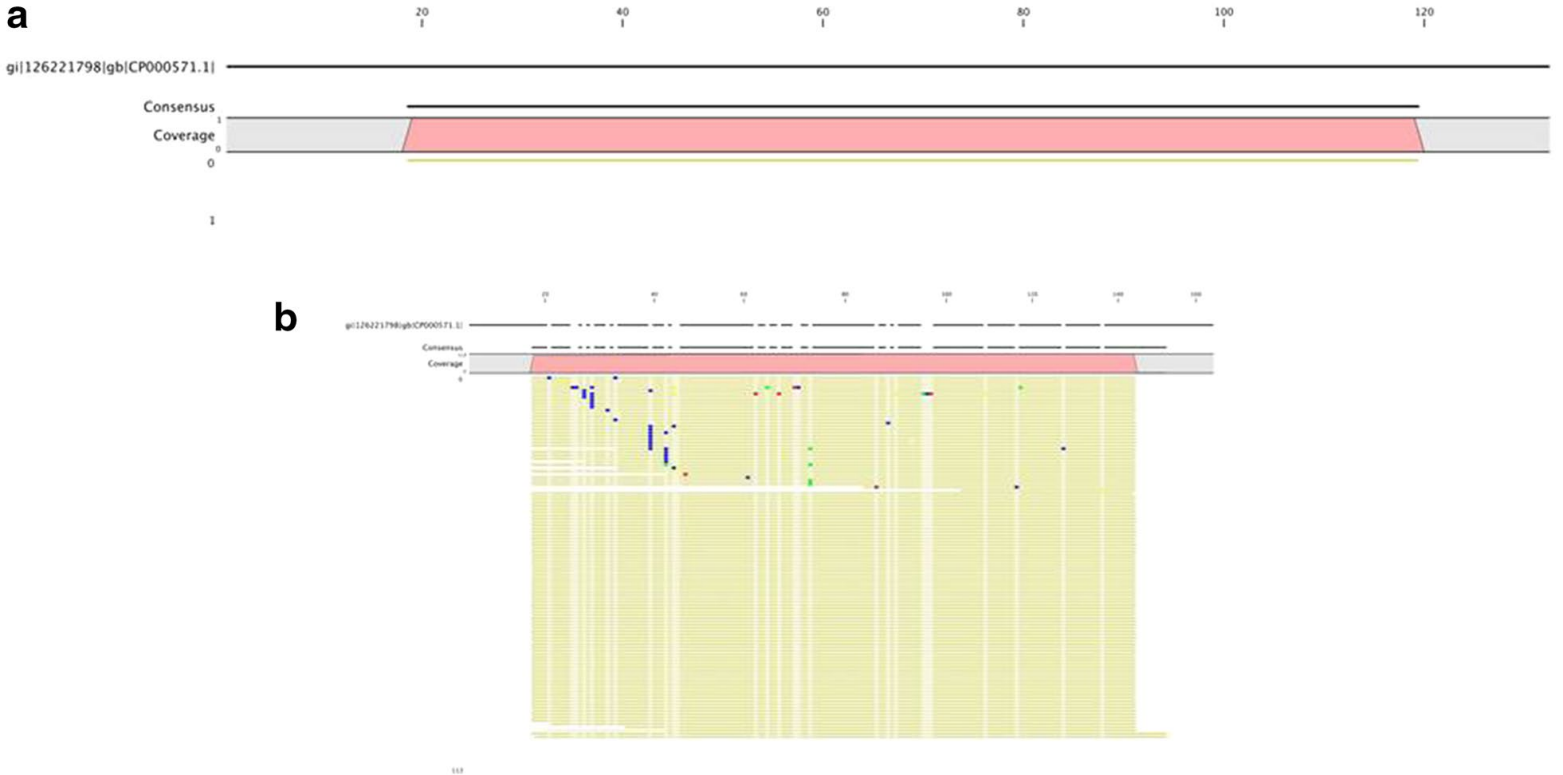
Read mapping against a representative amplicon from *B. pseudomallei* at 1e2 spike-in. (**a**), A single read resulting from a fragment library was mapped to the amplicon whereas in (**b**), 1367 reads from the corresponding AmpliSeq™ library were mapped to the same sequence. Areas of *different color* indicate base difference from the reference. *Blue* cytosine, *Red* adenine, *Green* thymine, *Yellow* guanine

Further, we observed that read depth was increased by orders of magnitude (Fig. 3) and an overwhelming majority of reads from the AmpliSeq™ libraries were able to be mapped to the highly-informative amplicons (Table 1). At the 1e2 spike level, the *T. maritima* positive control was the largest fraction of classified reads from the fragment library (Fig. 1), whereas the largest fraction of classified reads from the 1e4 spike fragment library was *F. tularensis* (Fig. 1). Interestingly, the second largest fraction of classified reads in the 1e3 spike samples was *T. maritima* even with a ten-fold increase of GEs versus that for the largest fraction, *F. tularensis*. This trend is also evident in 1e5 spike and 1e6 spike libraries. This suggests that bias exists in one of two steps in the fragment libraries: (1) Library construction or (2) Template amplification. There is also some preferential amplification evident in the AmpliSeq™ libraries as *F. tularensis* tended to be overrepresented in both the read mapping and taxonomic classifications for the lower spike levels. Additional file 1: Figure S1, Additional file 2: Figure S2, Additional file 3: Figure S3, Additional file 4: Figure S4 show details of the reads mapping to *B. anthracis*, *B. pseudomallei*, *Y. pestis*, and *F. tularensis* for all of the unamplified and amplified spike-in samples.

**Table 1:** Summary of read mapping to organism-specific amplicons for a single sequence run

Sample	Genome equivalents	Total reads mapped	Percent reads mapped (%)
1e6 Frag	1,000,000	7172	0.2
1e6 Amp	1,000,000	5,261,163	95.7
1e4 Frag	10,000	1338	0.02
1e4 Amp	10,000	5,781,522	97.7
1e3 Frag	1000	1021	0.01
1e3 Amp	1000	5,037,307	97.6
1e2 Frag	100	12,702	0.2
1e2 Amp	100	5,194,177	96.3

It is well known that competition for reagents can impact amplification of a given target(s) in multiplex PCR [27]. In order to investigate the impact of reducing the amount of positive amplification control, we created and sequenced two samples with *T. maritima* present at the same spike-in level as the organisms of interest (1e1Amp_1e1Tmar and 1e0Amp_1e0Tmar). As shown in Fig. 1 (far right), the fraction of reads assigned to *T. maritma* was reduced while the fraction of reads assigned to the spike-in organisms was increased. This was especially prominent in the samples at a spike in level of 1e1 GEs. In each case, the number of reads assigned to the spike-in organisms increased as compared to the same spike-in level containing a greater level of the positive amplification control (Additional file 1: Figure S1, Additional file 2: Figure S2, Additional file 3: Figure S3, Additional file 4: Figure S4). This demonstrates that the amount of positive control used may need to be considered depending on the desired limit of detection.

### Comparison of SNP typing

A common method of confident and reliable phylogenetic placement of a sample based on a set of sequence reads requires knowledge of single nucleotide polymorphisms (SNPs) present at informative loci. Sequence reads from all samples were compared to a proprietary database of all known SNPs for each spike-in organism, based on the multiple genomes available (Table 2). The number of SNP loci detected in the High (1e6) Fragment and Medium (1e4) Fragment libraries was greater than in any of the AmpliSeq™ samples. This is an expected result as the fragment libraries at these high concentrations (1e6, 1e4) potentially contain the whole genome, while the AmpliSeq™ libraries contain a small number of specifically-targeted loci (Table 3). There is also an expected large decrease in the number of SNPs detected at lower concentrations in the fragment libraries. Specifically there are no SNPs detected for *B. pseudomallei* or *B. anthracis* at the 10 GE level. In contrast, for AmpliSeq™ libraries, some loci are detected even at the very lowest spike in levels. The “predicted amplicons” in Table 2 indicates the theoretical maximum number of known SNP loci that should be covered in the AmpliSeq™ samples if exactly the predicted amplicons were sequenced. The point being made here is that the preferential amplification of these regions is what allows those known phylogenetically-informative SNP loci to be interrogated even at the lowest spike-in levels. Additional apparent SNPs could result from errors in the amplification or sequencing, or actual differences between the material spiked in and the associated reference genome. Polymorphisms and structural changes are known to occur in clonal populations [28].

**Table 2:** Number of aggregate SNPs in each sample by organism

Sample	*B. anthracis*	*B. pseudomallei*	*F. tularensis*	*Y. pestis*
1e6Frag	673	35,871	22,930	541
1e4Frag	354	9725	5542	372
1e3Frag	65	1403	3520	97
1e2Frag	18	135	82	15
1e1Frag	0	0	11	15
1e6Amp	109	2253	267	54
1e4Amp	45	1367	195	19
1e3Amp	30	1133	141	15
1e2Amp	32	963	166	13
1e1Amp	34	1030	155	16
1e0Amp	17	455	110	4
1e1Amp_1e1Tmar	34	950	147	13
1e0Amp_1e0Tmar	11	338	107	2
Total SNPs/genome	10,004	1,024,037	98,629	8934
Predicted SNPs	33	791	122	34

**Table 3:** 1e6 spike-in scheme and primer pairs per organism

Organism	Genome equivalents/sample	No of primer pairs
Human	3000	N/A
*Yersinia pestis*	1,000,000	99
*Francisella tularensis*	1,000,000	24
*Bacillus anthracis*	1,000,000	66
*Burkholderia mallei*	0	5
*Burkholderia pseudomallei*	1,000,000	124
*Salmonella enterica*	0	46
*Thermotoga maritima*	10,000	50

The reads cluster with the correct strain for *B. pseudomallei* and *F. tularensis* for all the samples with detected SNPs. However, this was not observed for *Y. pestis* or *B. anthracis.* This is likely due to the small number of SNPs present by chance in the amplicons chosen for identification of these organisms (33 and 34, respectively).

In general, neither fragment libraries nor AmpliSeq™ libraries reliably cluster with the correct strain (Additional file 5: Figure S5, Additional file 6: Figure S6, Additional file 7: Figure S7, Additional file 8: Figure S8). It should be noted that primers pairs were not designed to cover phylogenetically informative SNPs (although such targeting is of course possible). Rather, selected regions were chosen to be conserved at the species level. However, as concentrations drop, reliable SNP interrogation is possible only with the AmpliSeq™ libraries (Table 2).

## Discussion

These data demonstrate the difficulty involved in obtaining confident bacterial species identification from a complex mock human clinical sample via unbiased metagenomic sequencing. Employing targeted amplification provides a large increase in reads from the discriminating regions, allowing confident species identification to be made at very low spike-in levels. This demonstrates that a clear distinction needs to be made between the use of NGS as a novel discovery platform (where deep unbiased sequencing must be performed) and as a detector of known organisms/genes/SNPs (e.g., comparing against reference genome sequence information already available in databases).

The use of current benchtop NGS to confidently detect a panel of known pathogens at clinical or trace level in human or environmental samples will need to employ some effective form of background clutter mitigation, target capture (via microarray or other enrichment approaches), targeted amplification, or a combination of strategies. Dependence solely upon unbiased metagenomic benchtop sequencing will otherwise lack confidence that accurate species identification can be made of the known organisms that may be present at low levels. It should be noted that confident identification of important functional genes (e.g., critical anti-microbial resistance or virulence factors) cannot be achieved with current benchtop NGS for many types of metagenomic clinical samples [11] (other than relatively noncomplex clinical samples such as cerebrospinal fluid or urine, for which antimicrobial resistance prediction from unbiased NGS is more reasonable [29]). Without specific targeting for pathogens present at clinically-relevant levels and with 3000+ genes in a typical bacterium, the odds of the small set of unbiased reads that map to the pathogen hitting the handful of critical resistance or virulence genes are extremely low.

**Fig. 4:**
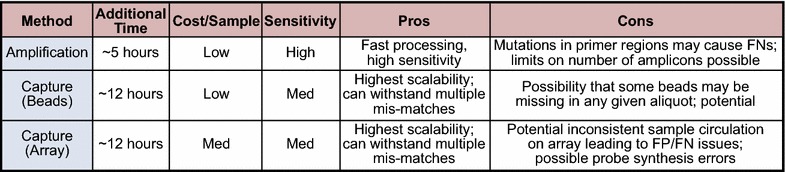
Relative comparison of different target enrichment methods. Targeted amplification and capture tradeoffs include time, cost, sensitivity, and scalability. Actual values will vary depending on sample type, details of targeting, and vendor-specific parameters

Although host removal techniques have been developed for rRNA, the more generalized concept of clutter mitigation has yet to be proven feasible for complex environmental samples. One such technique, Selective Whole Genome Amplification (SWGA), employs primers specific to a genome(s) of interest [30]. In principle, this technique is similar to the approach presented here. Although SWGA would produce sequence reads with broader coverage of the target genome, it has not yet been shown to be feasible for more than one genome. Additionally, methods for removal of host gDNA are still in development or are not yet proven to be efficacious in complex, metagenomic samples [31, 32]. This leaves capture enrichment techniques as the leading viable alternative to targeted amplification [33]. Array or bead-based capture enrichment [34] can employ sufficient probes to cover all the amplicons used in a targeted amplification panel; furthermore, capture approaches, in general, can scale much larger than current primer-based amplification panels available commercially [35]. The tradeoffs between the enrichment approaches center on factors such as limit of detection (large-scale capture enrichment arrays typically rely on some form of random amplification), the hybridization time required for capture at low levels, and the added cost of the capture step to the library prep process (Fig. 4). One possibility for a future project would be to perform a controlled comparison of the enhancement approaches in order to investigate the relative merits of each.

As both capture enrichment and targeted amplification techniques are continuing to advance rapidly it is premature to declare one technique to be better than the other for all applications. However, since capture enrichment arrays already can scale to over one million probes it is likely that this will be the best approach for very large scale targeting. It should also be noted that capture techniques will likely work better in cases with highly-degraded DNA where PCR often fails to work [36]. Similarly, since targeted amplification can provide lower levels of detection than random amplification against large genomic backgrounds with all else being equal, a lasting niche for this technique is also likely for applications targeting a moderate number of regions where sensitivity at low levels is vital. Future increases in NGS sequencing read lengths will be matched with larger amplicon targets, increasing the information content of targeted amplification even if the primer limits stay constant. A reasonable speculation is that there will continue to be multiple methods for enhancing sequencing of desired regions.

The targeted amplification technique demonstrated here can be aimed at multiple levels of resolution. Amplicons can be selected to identify organisms, as shown here, to identify genes of interest (e.g. known virulence and/or anti-microbial resistance factors), or to identify SNPs of interest. For example, it would be possible to create an AmpliSeq™ panel that specifically targeted key genes or sufficient SNPs to achieve a specified level of phylogenetic resolution for a set of microbial species, up to the limit of the maximum allowed number of primer pairs for the AmpliSeq™ product. This means that if we designed an AmpliSeq™ panel to cover enough phylogenetically informative SNPs by ensuring that the SNPs on predicted amplicons are sufficient to recapitulate the phylogeny based on all SNPs or whole genome alignments, we should get accurate genotyping and fine level phylogenetic classification even at very low concentrations of the target. It would even be possible to mix amplicons that query organisms, genes, and SNPs in the same panel, subject to the limit of primer pairs.

## Conclusions

Our pilot study of a targeted amplification panel focused on detecting several biothreat agents against a human DNA background demonstrated that confident species determination could be made at least 2 orders of magnitude of spike-in level below that of an unbiased library preparation. Owing to the design of the primers in the targeted amplification panel, all reads from those amplicons are guaranteed to be informative at the species-resolution level. (Since variations in targeted organisms could cause individual primers to fail, we designed multiple highly-discriminating amplicons to provide redundancy.) This contrasts to the unbiased sequencing, where any reads mapping to the pathogen come from random locations, many of which are common to multiple species at higher taxonomic levels of resolution. Thus, the use of targeted amplification on a bench-top NGS platform provides a workable approach to confident and information-rich identification of a set of known pathogens from a complex sample when alternatives such as culturing or ultra-deep NGS are not feasible options.

Although we demonstrated our approach using a relatively small biothreat agent panel of 467 amplicon targets, the targeted amplification technique could obviously be extended to other applications such as human or animal health, food safety, vaccine or biological product safety, contamination monitoring for manufacturing processes, or any application where it is important to know whether a set of known pathogens are present. It should be clear that targeting highly-discriminating regions of a whole genome provides much more resolution power than amplifying and sequencing only the 16S rRNA gene, although that process still remains a viable choice for performing a broad census of bacteria present; including some that may not yet have whole genomes available. Finally, although AmpliSeq™ is designed for use on Ion platforms, it is possible that it may be adapted for use with other sequencing technologies or alternative solutions may soon become available. Additionally, we have not tested the efficacy of the much larger AmpliSeq™ panels that can be supported by the current product.

## Methods

### Sample preparation

Samples were created in accordance with the manufacturers’ recommendations for nucleic acid mass. Briefly, a base mix of *Bacillus anthracis* (Sterne), *Yersinia pestis* (Harbin), *Francisella tularensis* (LVS) and *Burkholderia pseudomallei* (strain 9) genomic DNA (gDNA) were created such that the number of genome equivalents (GE) of each gDNA was 2× the final concentration of the 1e6 Spike mix (refer to Table 3). Ten nanograms (3000 GE) of human gDNA (Clonetech Laboratories; Mountain View, CA, USA) and 10,000 GE of *Thermotoga maritima* strain MSB8 gDNA were added in amounts that were constant for each sample. *T. maritima* (a deep sea vent thermophile) was used here as an amplification control. Final sample volumes were 20 µL. Samples were given designations based on the pathogen GEs spiked as follows: (1e6), (1e5), (1e4), (1e3), and (1e2). Pathogen spike-ins of 10 and 1 (1e1Amp, 1e0Amp) GE were also sequenced for the AmpliSeq™ libraries, with *T. maritima* at 10,000 GE or at the same 10 or 1 GE as the pathogens (1e1Amp_1e1Tmar, 1e0Amp_1e0Tmar). Copy numbers calculations for all spike-in organisms were based on the published genome and plasmid sizes in base pairs using NCBI accession numbers. The relevant accession numbers are as follows: *B. anthracis* NC_007530 (chromosome), NC_007322 (pXO1); *F. tularensis* NC_007880.1; *Y. pestis* NC_003143.1 (chromosome), NC_003132.1 (pPCP1), NC_003131.1 (pPCD1), and NC_003134.1 (pMT1); and *B. pseudomallei* NC_006350.1 (chromosome 1) and NC_006351.1 (chromosome 2).

### Library preparation and sequencing

AmpliSeq™ libraries were constructed with the Ion AmpliSeq™ library protocol and the following parameters: 2× primer mix, 6 µL of sample and 16 PCR cycles. Fragment libraries were constructed by following the suggested Ion PGM protocol and 6 µL of sample. All libraries were quality checked using the Agilent BioAnalyzer and quantitated using the Ion Library Quantitation Kit. Template was diluted to target 10–30 % enriched beads and clonally amplified using Ion PGM OT2 400 kit. Enriched beads were sequenced using the Ion PGM 400 Sequencing kit on an Ion 318™ chip at default instrument parameters. Each library was sequenced separately. Sequence run metrics are summarized in Additional file 9: Table S1.

### AmpliSeq™ panel design

A total of 9799 organism-specific amplicons created using custom software [6] were submitted to Life Technologies for down-selection and primer design using the Ion AmpliSeq™ strategy and the Ion AmpliSeq™ software. All amplicons were from genomic regions of the study organisms that maximally distinguish them at the species level from all other microbial organisms with genomes publically available at the time of design. Following several iterative rounds of primer optimization and removal of overlapping amplicons, a panel of 467 primer pairs was chosen for the pilot panel (Table 3). Amplicon sequences are available upon request. Primer information is not supplied by Life Technologies.

### Bioinformatic analysis

#### Reference mapping

Sequence read mapping was performed using CLC Genomics Workbench v6.5 (CLC Inc, Aarhus, Denmark). CLC Reference Mapper was run with default settings (Insertion cost = 3, Deletion cost = 3, Mismatch cost = 2, Length fraction = 0.5 and similarity fraction = 0.8). Mapping of all sequence reads was performed against the amplicon sequences that comprise the current panel (Table 3) and/or the available NCBI reference genome for the spike in organisms.

#### Taxonomic classification of sequence reads

Sequence reads were trimmed and quality filtered prior to analysis using LMAT [37]. LMAT uses a custom *k*-mer (sequence strings of length *k*) database of all draft and finished microbial genomes to classify sequence reads. Each read is mapped to the genome(s) to which it best corresponds, and then a score is computed to indicate the confidence for each mapped genome being present. Data were visualized using MEGAN [38]. For comparisons between samples, taxa were reported for which the minimum read score averaged across reads was ≥1 and there were at least 50 reads. Additionally, read counts were normalized as a fraction of the total reads in that run.

## Availability of supporting data

Supporting data may be available upon request.

## Additional files

10.1186/s13104-015-1530-0 Sequence reads classified as *B. anthracis*. Sequence reads resulting from the indicated samples were classified using LMAT with the complete genome database as indicated in the “Methods”.
10.1186/s13104-015-1530-0 Sequence reads classified as *B. pseudomallei*. Sequence reads resulting from the indicated samples were classified using LMAT with the complete genome database as indicated in the “Methods”.
10.1186/s13104-015-1530-0 Sequence reads classified as *F. tularensis*. Sequence reads resulting from the indicated samples were classified using LMAT with the complete genome database as indicated in the “Methods”.
10.1186/s13104-015-1530-0 Sequence reads classified as *Y. pestis*. Sequence reads resulting from the indicated samples were classified using LMAT with the complete genome database as indicated in the “Methods”.
10.1186/s13104-015-1530-0 Taxonomic comparison of sequence reads at 1e1 GE spike-in. Sequence reads resulting from the indicated samples were classified using LMAT with the complete genome database as indicated in the Materials and Methods. Data were visualized using MEGAN. Taxa are reported for which the minimum read score averaged across reads was ≥ 1 and there were at least 50 reads.
10.1186/s13104-015-1530-0 Taxonomic comparison of sequence reads at 1e2 GE spike-in. Sequence reads resulting from the indicated samples were classified using LMAT with the complete genome database as indicated in the Materials and Methods. Data were visualized using MEGAN. Taxa are reported for which the minimum read score averaged across reads was ≥ 1 and there were at least 50 reads.
10.1186/s13104-015-1530-0 Taxonomic comparison of sequence reads at 1e3 GE spike-in. Sequence reads resulting from the indicated samples were classified using LMAT with the complete genome database as indicated in the Materials and Methods. Data were visualized using MEGAN. Taxa are reported for which the minimum read score averaged across reads was ≥ 1 and there were at least 50 reads.
10.1186/s13104-015-1530-0 Taxonomic comparison of sequence reads at 1e4 GE spike-in. Sequence reads resulting from the indicated samples were classified using LMAT with the complete genome database as indicated in the Materials and Methods. Data were visualized using MEGAN. Taxa are reported for which the minimum read score averaged across reads was ≥ 1 and there were at least 50 reads.
10.1186/s13104-015-1530-0 Summary of sequencing statistics.
