# Supplementary figures and images for: Targeted amplification for enhanced detection of biothreat agents by next-generation sequencing
Source: BMC Res Notes. 2015 Nov 16;8:682. doi: 10.1186/s13104-015-1530-0 (PMC4647626; doi:10.1186/s13104-015-1530-0)

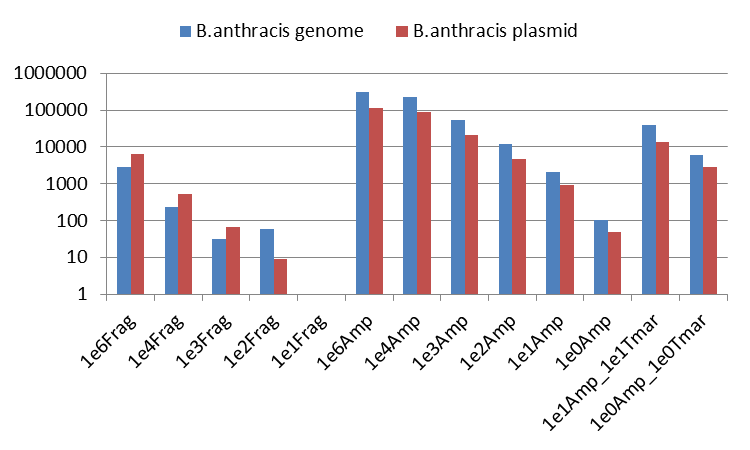

Supplement: Supplementary file 1 — 10.1186/s13104-015-1530-0 Sequence reads classified as B. anthracis. Sequence reads resulting from the indicated samples were classified using LMAT with the complete genome database as indicated in the “Methods”. [file 13104_2015_1530_MOESM1_ESM.png]

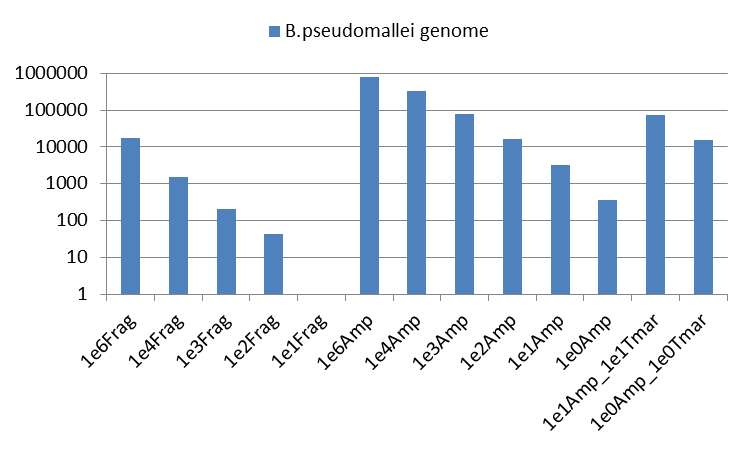

Supplement: Supplementary file 2 — 10.1186/s13104-015-1530-0 Sequence reads classified as B. pseudomallei. Sequence reads resulting from the indicated samples were classified using LMAT with the complete genome database as indicated in the “Methods”. [file 13104_2015_1530_MOESM2_ESM.png]

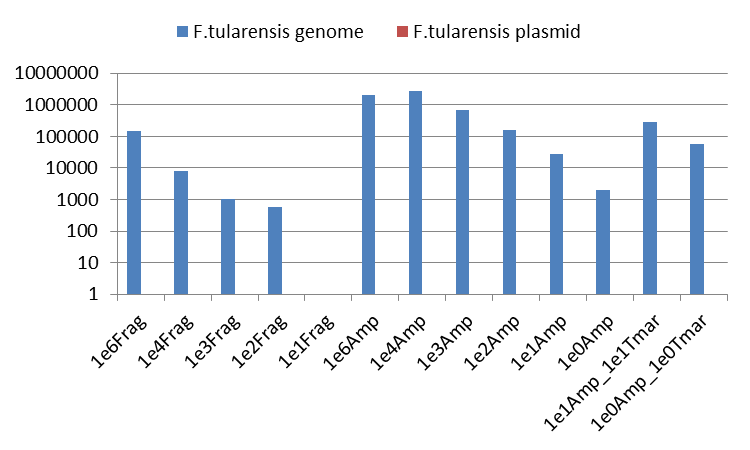

Supplement: Supplementary file 3 — 10.1186/s13104-015-1530-0 Sequence reads classified as F. tularensis. Sequence reads resulting from the indicated samples were classified using LMAT with the complete genome database as indicated in the “Methods”. [file 13104_2015_1530_MOESM3_ESM.png]

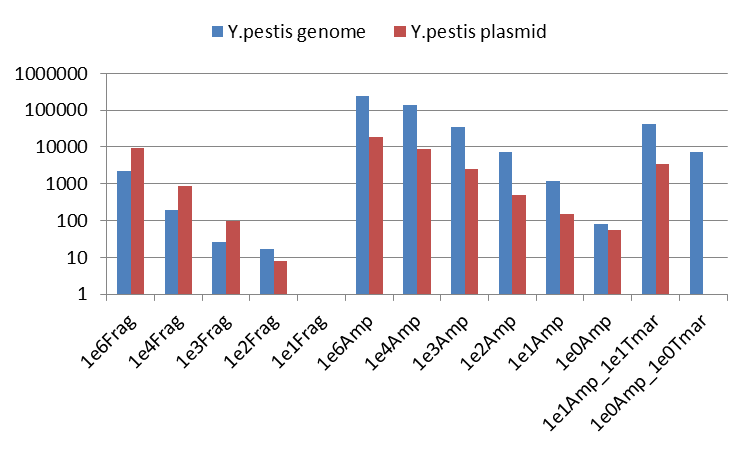

Supplement: Supplementary file 4 — 10.1186/s13104-015-1530-0 Sequence reads classified as Y. pestis. Sequence reads resulting from the indicated samples were classified using LMAT with the complete genome database as indicated in the “Methods”. [file 13104_2015_1530_MOESM4_ESM.png]

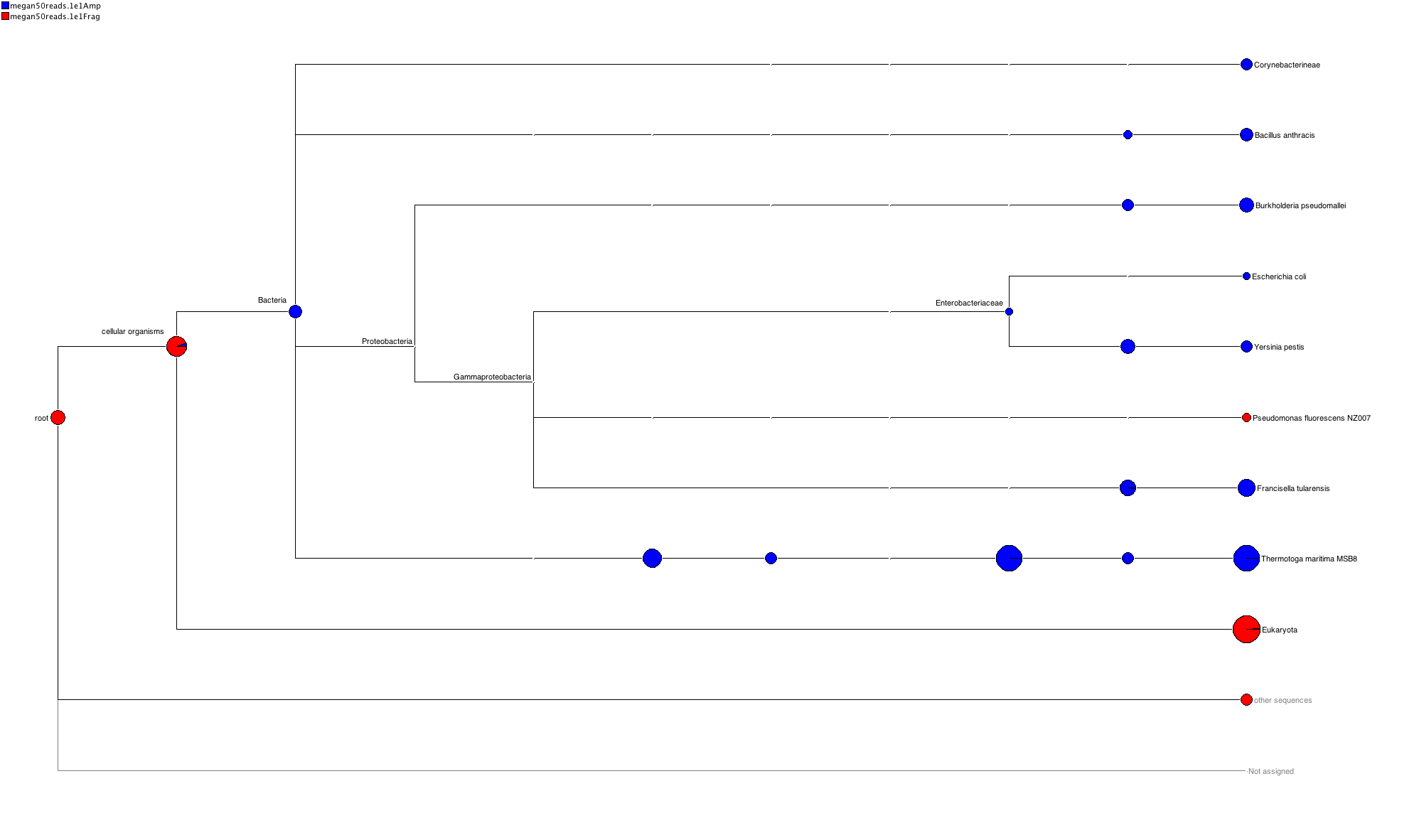

Supplement: Supplementary file 5 — 10.1186/s13104-015-1530-0 Taxonomic comparison of sequence reads at 1e1 GE spike-in. Sequence reads resulting from the indicated samples were classified using LMAT with the complete genome database as indicated in the Materials and Methods. Data were visualized using MEGAN. Taxa are reported for which the minimum read score averaged across reads was ≥ 1 and there were at least 50 reads. [file 13104_2015_1530_MOESM5_ESM.png]

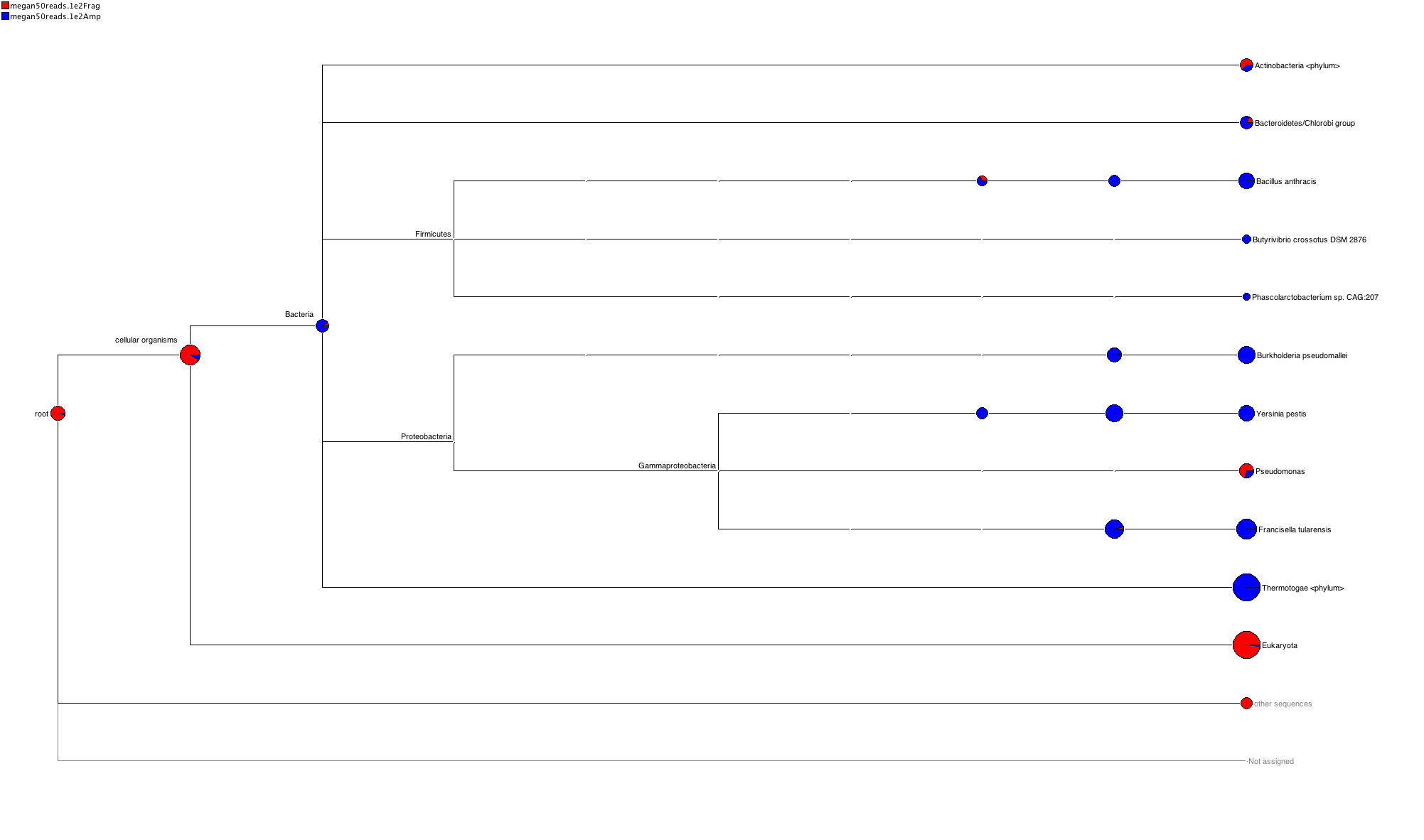

Supplement: Supplementary file 6 — 10.1186/s13104-015-1530-0 Taxonomic comparison of sequence reads at 1e2 GE spike-in. Sequence reads resulting from the indicated samples were classified using LMAT with the complete genome database as indicated in the Materials and Methods. Data were visualized using MEGAN. Taxa are reported for which the minimum read score averaged across reads was ≥ 1 and there were at least 50 reads. [file 13104_2015_1530_MOESM6_ESM.png]

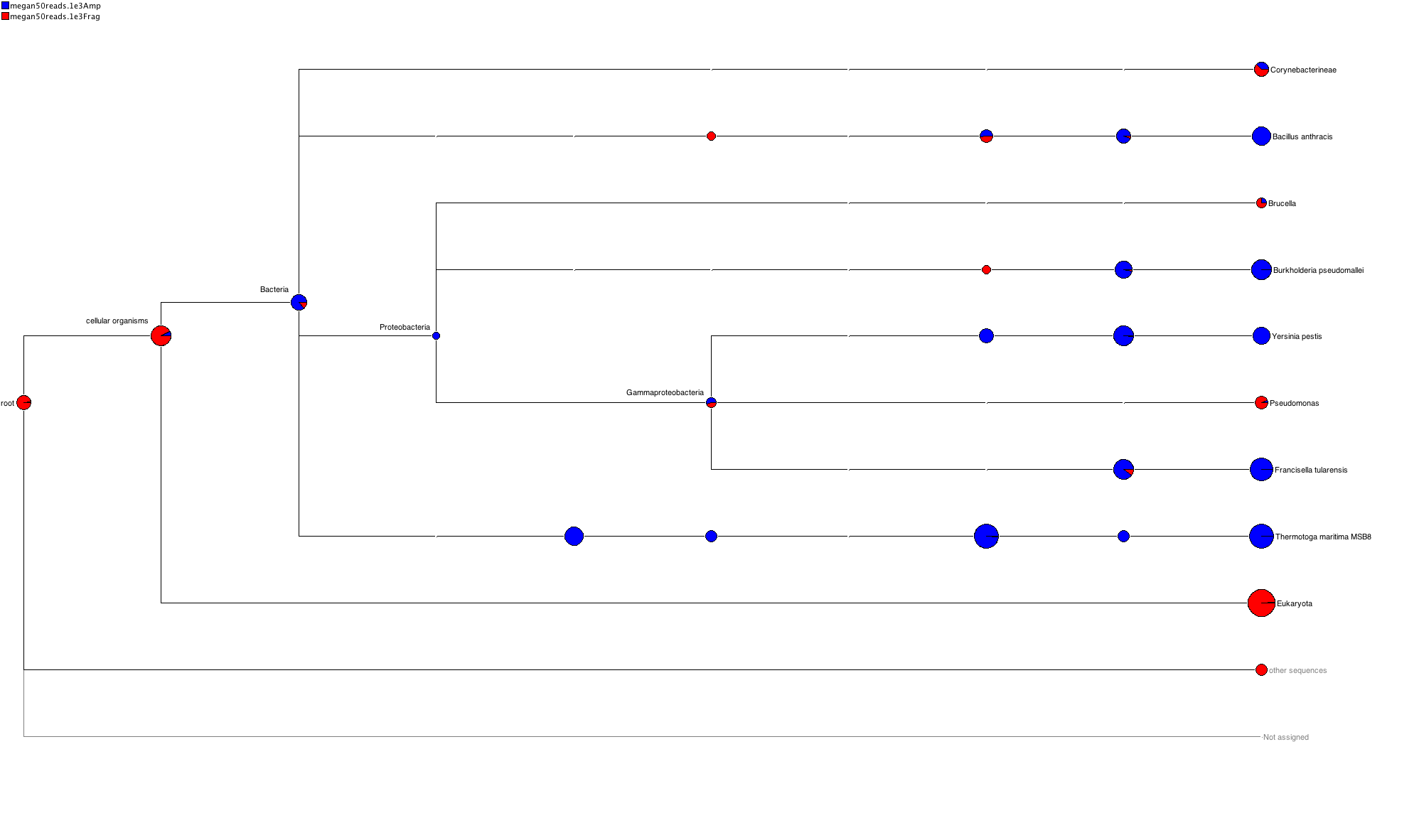

Supplement: Supplementary file 7 — 10.1186/s13104-015-1530-0 Taxonomic comparison of sequence reads at 1e3 GE spike-in. Sequence reads resulting from the indicated samples were classified using LMAT with the complete genome database as indicated in the Materials and Methods. Data were visualized using MEGAN. Taxa are reported for which the minimum read score averaged across reads was ≥ 1 and there were at least 50 reads. [file 13104_2015_1530_MOESM7_ESM.png]

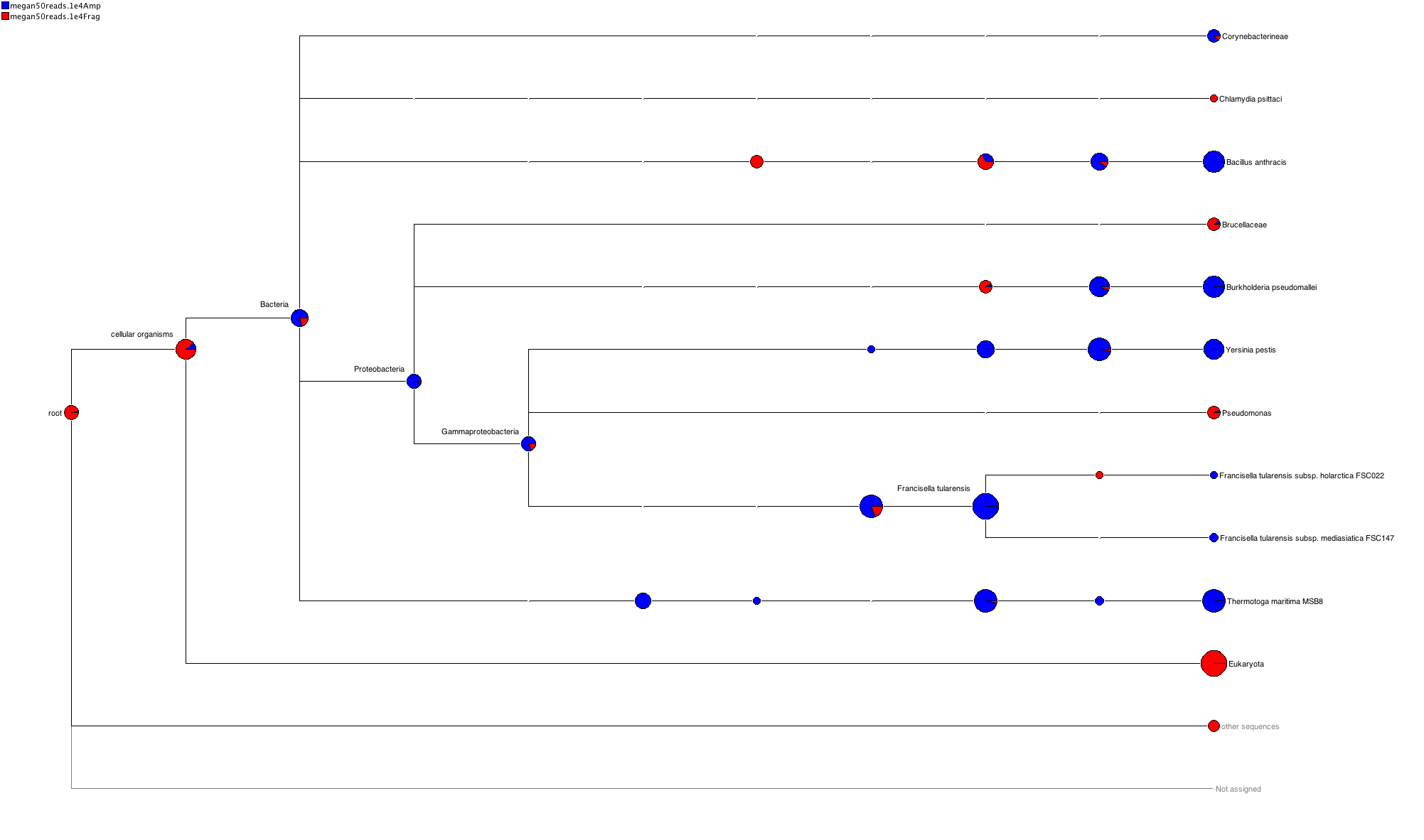

Supplement: Supplementary file 8 — 10.1186/s13104-015-1530-0 Taxonomic comparison of sequence reads at 1e4 GE spike-in. Sequence reads resulting from the indicated samples were classified using LMAT with the complete genome database as indicated in the Materials and Methods. Data were visualized using MEGAN. Taxa are reported for which the minimum read score averaged across reads was ≥ 1 and there were at least 50 reads. [file 13104_2015_1530_MOESM8_ESM.png]
