# Supplementary material for: Targeted amplification for enhanced detection of biothreat agents by next-generation sequencing
Source: BMC Res Notes. 2015 Nov 16;8:682. doi: 10.1186/s13104-015-1530-0 (PMC4647626; doi:10.1186/s13104-015-1530-0)
Supplement: Supplementary file 9 — 10.1186/s13104-015-1530-0 Summary of sequencing statistics. [file 13104_2015_1530_MOESM9_ESM.docx]

**Supplemental Table 1 – Summary of sequencing statistics**

| **Sample** | **Total Reads** | **Mean Length (bp)** | **Total Bases** | **Q20 Bases** |
| --- | --- | --- | --- | --- |
| 1e6 Frag | 3.52 M | 244 | 860 Mb | 678 Mb |
| 1e6 Amp | 5.36 M | 158 | 879 Mb | 736 Mb |
| 1e4 Frag | 4.58 M | 267 | 1.23 Gb | 1.02 Gb |
| 1e4 Amp | 5.92 M | 134 | 797 Mb | 716 Mb |
| 1e3 Frag | 5.29 M | 272 | 1.44 Gb | 1.20 Gb |
| 1e3 Amp | 5.16 M | 128 | 664 Mb | 609 Mb |
| 1e2 Frag | 5.29 M | 245 | 1.30 Gb | 1.06 Gb |
| 1e2 Amp | 5.39 M | 129 | 696 Mb | 624 Mb |
